# Burkitts’s lymphoma – an atypical presentation

**DOI:** 10.1186/1471-2431-12-113

**Published:** 2012-08-06

**Authors:** Farah Ziade, Nicolas von der Weid, Maja Beck-Popovic, Andreas Nydegger

**Affiliations:** 1 Pediatric Gastroenterology Unit, Department of Pediatrics, University of Lausanne, Centre Hospitalier Universitaire Vaudois, Rue du Bugnon 46, CH-1011, Lausanne, Switzerland; 2 Pediatric Hematology/Oncology Unit, Department of Pediatrics, University of Lausanne, Centre Hospitalier Universitaire Vaudois, Rue du Bugnon 46, CH-1011, Lausanne, Switzerland

**Keywords:** Burkitt’s lymphoma, Gastric, Stomach, Adolescent, Epstein barr virus, Krukenberg tumor

## Abstract

**Background:**

In female adolescents and young adults, malignancies of the genital tract are the most frequent type of cancer, closely followed by Hodgkin’s and non-Hodgkin’s lymphomas.

**Case Presentation:**

We report an unusual case of sporadic Burkitt’s lymphoma (BL) presenting with massive bilateral ovarian infiltration, peritoneal carcinomatosis and diffuse nodular lesions of the stomach and the intestine mimicking Krukenberg tumor. Diagnostic biopsies were obtained by endoscopy of the upper gastrointestinal tract. With intensive chemotherapy, complete remission was rapidly achieved, without life-threatening tumor lysis syndrome.

**Conclusion:**

Besides metastatic gastric adenocarcinoma, BL is an important differential diagnosis in adolescents presenting with Krukenberg tumor.

## Background

Cancer in adolescents and young adults is 2.7 times more common than cancer occurring during the first 15 years of life. In females aged between 15 to 29 years old, malignancies of the genital tract are the most frequent type of cancer (18%), closely followed by lymphomas (17%) [1]. Hodgkin lymphomas account for two–thirds of those cases. The remainder of patients present one of the four subtypes of non-Hodgkin lymphoma (NHL): diffuse large B-cell lymphoma, Burkitt’s lymphoma (BL), lymphoblastic lymphoma or anaplastic large cell lymphoma.

## Case presentation

**Figure 1:**
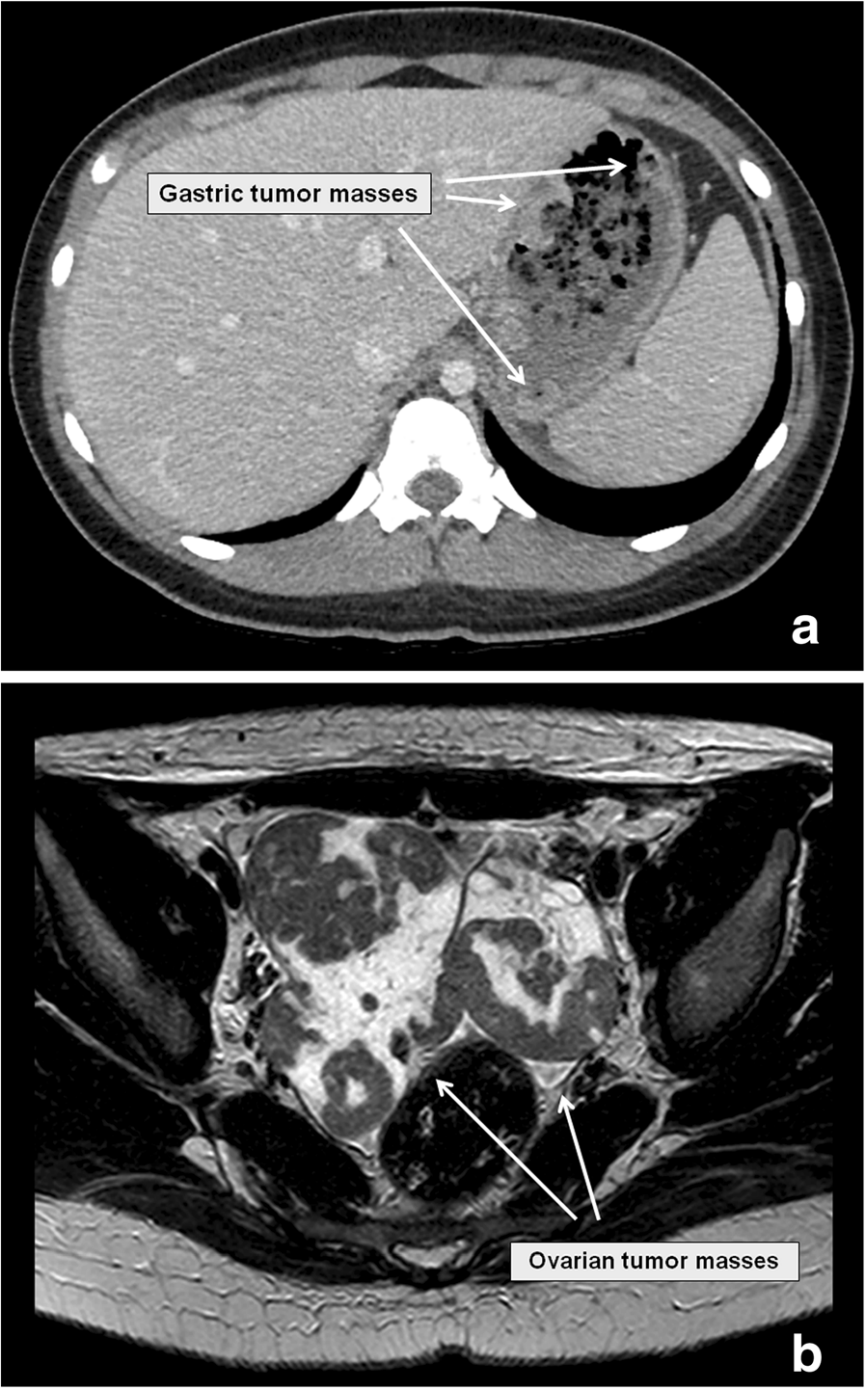
Abdominal CT scan with gastric tumor (a) and MRI showing bilateral ovarian masses (b).

A 15 year old girl presented to our outpatient clinic with a one month history of abdominal pain, vomiting, nausea, early satiety and weight loss of 3 kg. Clinical examination showed pallor and pitting oedema. No palpable abdominal mass was detected clinically but ultrasonography revealed huge bilateral ovarian masses, suggestive of Krukenberg tumor. Initial laboratory findings showed normocytic, normochromic anaemia with a haemoglobin of 93 g/L, a white blood cell count (WBC) of 16.6 x10^3^/mm^3^, elevated platelets of 484,000/mm^3^ (neutrophils, lymphocytes and blood film were normal). Total serum proteins as well as albumin were slightly reduced with 43 g/L and 23 g/L respectively, with normal liver and renal function tests. Serum lactate dehydrogenase (LDH) level was 334 UI/L, uric acid 334 μmol/l and ferritin 36 μg/l confirming absence of tumor lysis. Both alpha foeto-protein (AFP) and beta human chorionic gonadotropin (βHCG) were within normal range. The abdominal CT and MRI showed bilateral ovarian masses, peritoneal carcinomatosis, ascites and diffuse and nodular thickening of the gastric and intestinal wall (Figure 1).

**Figure 2:**
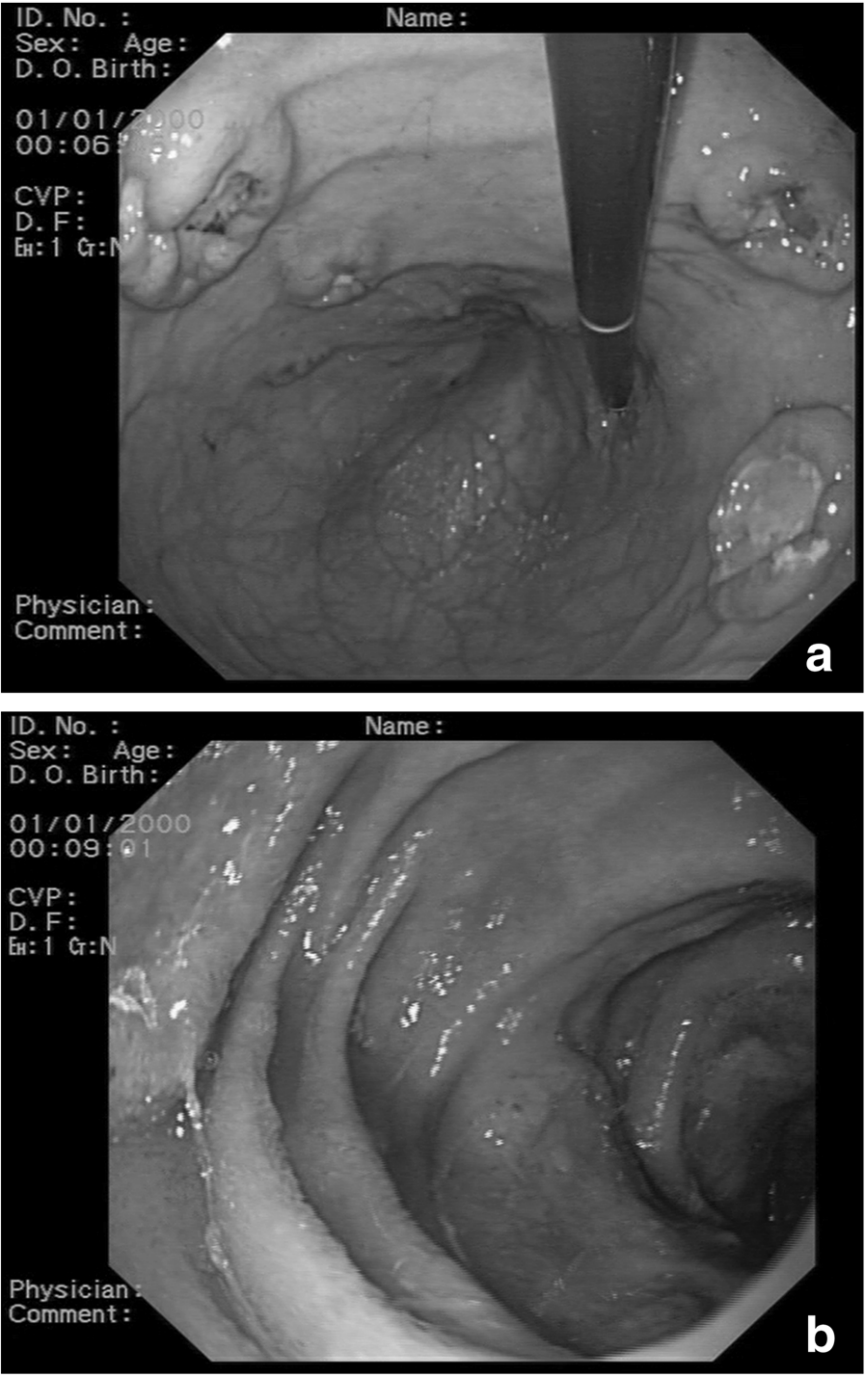
Gastric (a) and jejunal (b) lesions on upper endoscopy.

Suspecting obstructive endoluminal masses, an upper endoscopy was performed and revealed multiple, large (2 to 3 cm in diameter), raised ulcerated tumors involving the stomach, as well as multiple similar lesions throughout the duodenum and jejunum (Figure 2); multiple biopsies were taken from several lesions for further analysis. Histological examination showed diffuse wall infiltration by a medium sized, monotonous, atypical lymphoid cell population with scanty basophilic cytoplasm and a characteristic starry-sky pattern. On immunohistochemistry tumor cells showed positive staining for B-cell-associated antigens (CD 20, CD 10), B cell lymphoma 6 protein (Bcl6), paired box gene 5(PAX 5), surface IgM and IgD as well as monoclonal Kappa light chains. They were negative for B cell leukemia/lymphoma 2(Bcl2) and T-cell marker (CD5). These morphological and immunophenotypical features were consistent with Burkitt’s lymphoma, classified as stage III, Group B according to Murphy Stages. EBV encoded small RNA (EBER) in situ hybridisation showed an 80% consistency with Epstein Barr Virus (EBV). Finally, the typical reciprocal chromosomal translocation involving the proto-oncogene c-MYC on chromosome 8 and the immunoglobulin-gene heavy chain locus on chromosomes 14 [t(8;14)] was found in the tumor cells. Bone marrow aspirate and biopsy, as well as cerebrospinal fluid (CSF) were normal.

A nasojejunal feeding tube was inserted because of subtotal intestinal obstruction by a tumoral mass at the level of the duodenal bulb. The patient was started on chemotherapy according to POG 9917 protocol, including dexamethasone, methotrexate, cyclophosphamide, vincristin, cytarabin and doxorubicin; intrathecal cytarabin and methotrexate were administered as CNS prophylaxis. To prevent massive tumor lysis syndrome, hyperhydration and two doses of rasburicase were administered before and during the first cycle of chemotherapy. Interestingly, tumor lysis could be easily controlled and a rapid resolution of clinical signs was observed.

According to the protocol, chemotherapy was discontinued after 4 months. An upper endoscopy with biopsies was performed and confirmed remission with absence of lesions.

## Discussion and conclusion

To the best of our knowledge, this is the first pediatric report of Burkitt’s lymphoma mimicking Krukenberg tumor with synchronous involvement of stomach and ovary, even though ovarian or gastric implants of Burkitt´s lymphoma are a common finding. Similar reports have only been described in adult female patients, where gastric adenocarcinoma was the underlying disease [2,3].

Krukenberg tumor is generally defined as an ovarian carcinoma that contains a significant component of mucin-filled signet-ring cells typically lying within a cellular stroma derived from the ovarian stroma. It refers to a malignancy with metastasis to other organs, classically the gastrointestinal tract. Today, almost all Krukenberg tumors are thought to be metastatic signet-ring cell carcinoma in the ovary of gastric origin, although rare examples have been interpreted as primary.

Lymphomas represent 26% of all cancers in 15–29 year olds in Europe and the USA. In the 15–19 year-old group, diffuse large B-cell Lymphomas account for the largest proportion of NHL cases (37%) followed by BL (21%) [4].

Patients with gastrointestinal BL may present with abdominal pain or distension, gastrointestinal bleeding or intestinal obstruction resulting from direct compression of the lumen by an expanding mass or by an intussusception triggered by the intraluminal projection of the tumor mass [5-7]. These acute abdominal symptoms often lead to emergency laparotomies before a diagnosis of Burkitt’s lymphoma can be made [8].

Spontaneous intestinal perforation is an uncommon complication of BL, even though previously reported in children [9]. Possible explanations for spontaneous gastrointestinal perforation are tumor necrosis, immune suppression and protein malnutrition [9,10]. Tumor necrosis is probably the most likely reason as perforation usually occurs during or after the first cycle of intensive chemotherapy. Epigastric pain is the first and most common symptom of gastric perforation. A high degree of suspicion should be present at all times, as early diagnosis and emergency surgery are crucial in saving life in the setting of such a complication [11].

Burkitt’s and B-cell lymphomas in childhood have an excellent overall prognosis regardless of the location (except for primary central nervous system lymphoma), especially when treated with contemporary chemotherapy protocols [12].

Burkitt lymphoma has to be considered as differential diagnosis in adolescent girls presenting with apparent Krukenberg tumors. Prognosis remains excellent with precise histological and molecular diagnosis, early treatment and careful monitoring and prevention of tumor lysis.

## Competing interests

The authors declare that they have no competing interests.

## Authors’ contributions

FZ drafted the manuscript and collected data related to the subject; NVDW was involved in revising critically the manuscript and helped to draft the manuscript; MBP made substantial contributions in conception and design of the manuscript. AN participated in the design of the manuscript and the coordination. All authors read and approved the final manuscript

## Pre-publication history

The pre-publication history for this paper can be accessed here:

http://www.biomedcentral.com/1471-2431/12/113/prepub
